# Differential effects of PGAM5 knockout on high fat high fructose diet and methionine choline-deficient diet induced non-alcoholic steatohepatitis (NASH) in mice

**DOI:** 10.1186/s13578-023-01095-3

**Published:** 2023-08-21

**Authors:** Li Li, Chengcheng Guo, Yue Yu, Lu Tie, Guotao Lu, Feng Liu, Xueyao Han, Linong Ji, Xiantong Zou

**Affiliations:** 1 Department of Endocrinology and Metabolism, Peking University People’s Hospital, No.11 Xizhimen South Street, Xicheng District, Beijing, 100044 People’s Republic of China; 2 Department of Pharmacology, School of Basic Medical Sciences, Peking University Health Science Center, Beijing, 100191 China; 3 Department of Gastroenterology, Affiliated Hospital of Yangzhou University, Yangzhou University, Yangzhou, 225001 China; 4 Beijing Key Laboratory of Hepatitis C and Immunotherapy for Liver Diseases, Beijing International Cooperation Base for Science and Technology on NAFLD Diagnosis, Peking University People’s Hospital, Peking University Hepatology Institute, Beijing, 100044 China; 5 Present Address: Department of Nutrition, Beijing Chaoyang Hospital, Capital Medical University, Beijing, 100020 China

**Keywords:** PGAM5, NASH, Obesity, Mitochondria, Anti-oxidant

## Abstract

**Background:**

Phosphoglycerate mutase 5 (PGAM5), a phosphatase involved in mitochondrial homeostasis, is reported to be closely related to the metabolic stress induced by high-fat diet (HFD) or cold. In this study, we aimed to investigate the effects of PGAM5 on hepatic steatosis, inflammation and fibrosis in nonalcoholic steatohepatitis (NASH).

**Methods and results:**

We generated PGAM5 global knockout (GKO) mice and their wildtype (WT) littermates using CRISPR/CAS9. The mice were fed with a high fat high fructose (HFHF) diet for 12 weeks or a methionine choline-deficient (MCD) diet (methionine choline supplemented (MCS) as control) for 6 weeks. Hepatic PGAM5 expression was up-regulated in humans with NASH and WT mice fed with HFHF and MCS, and reduced in WT mice fed with MCD diet. In HFHF-fed mice, GKO had reduced body weight, hepatic triglyceride (TG) content and serum transaminase along with decreased hepatic pro-inflammatory and pro-fibrotic responses compared with their WT control. GKO had increased expression of antioxidative gene glutathione peroxidase-6 (GPX6) and activation of mammalian target of rapamycin (mTOR). In mice fed with MCS diet, GKO significantly increased serum TNF-α and IL-6 and decreased hepatic GPX6 mRNA expression. There was no difference in hepatic steatosis, inflammation or fibrosis between GKO and WT mice fed with MCD diet. We investigated the role of PGAM5 deficiency in a variety of cell types. In differentiated THP-1 cells, PGAM5 silencing significantly increased pro-inflammatory cytokine secretion and decreased antioxidative proteins, including nuclear factor erythroid 2- related factors (NRF2), heme oxygenase-1 (HO-1) and GPX6 without affecting mTOR activity. In HepG2 cells with steatosis, PGAM5 knockdown reduced insulin sensitivity, increased mTOR phosphorylation and reduced the expression of NRF2, catalase (CAT), HO-1 and GPX6. Conversely, PGAM5 knockdown reduced TG accumulation, increased insulin sensitivity, and increased antioxidative genes in 3T3-L1 cells, despite the up-regulation in mTOR phosphorylation.

**Conclusions:**

PGAM5-KO relieved hepatic steatosis and inflammation in HFHF model, promoted inflammation in MCS-fed mice and had no effects on the MCD-fed model. The distinct effects may be owing to the different effects of PGAM5-KO on anti-oxidative pathways in energy-dependent, possible involves mTOR, and/or cell type-dependent manner. Our findings suggest that PGAM5 can be a potential therapeutic target for NASH.

**Supplementary Information:**

The online version contains supplementary material available at 10.1186/s13578-023-01095-3.

## Background

Non-alcoholic fatty liver disease (NAFLD) is the most common chronic liver disease affecting around one quarter of the world’s population [1]. NAFLD comprises a wide spectrum of conditions ranging from simple steatosis to non-alcoholic steatohepatitis (NASH) and ultimately fibrosis, cirrhosis and hepatocellular carcinoma [2]. NASH is one of the advanced stages of NAFLD which is characterized by histological inflammation and early fibrosis, and the progression from simple steatosis to NASH is highly heterogeneous. Metabolic disorders, insulin resistance, oxidative stress, mitochondrial dysfunction, and endoplasmic reticulum (ER) stress may contribute to triggering hepatic inflammation and stimulating the progression to NASH [3]. Inflammatory cells such as macrophages play as a key ‘regulator’ in the process of hepatic fibrosis progression or regression [4]. Currently, there is no drug approved by the FDA for NASH treatment [5], although evidence of some drugs impeding the progression of hepatic inflammation is accumulating. Searching for novel therapeutic targets for NASH is necessary to satisfy the unmet clinical demand.

Among numerous targets investigated, mitochondrial proteins have gained substantial attention since they are related to energy metabolism and oxidative stress, which are key processes of NASH development [6]. Phosphoglycerate mutase family member 5 (PGAM5) is a protein phosphatase that resides in the mitochondria and regulates many biological processes, including cell death, mitophagy, and immune responses [7, 8]. Recent studies reported that knockout of PGAM5 showed resistance against cold and fasting-induced metabolic stress and high-fat-diet (HFD) -induced obesity, indicating PGAM5 may act as a metabolic regulator [9]. Recently, He et al. reported that PGAM5 may stimulate acute liver injury by mediating programmed hepatocyte death [10]. In other animal and cell models, PGAM5 seemed to stimulate pro-inflammatory process. Silencing PGAM5 reduced NKT cell activation, impaired IL-1β secretion in bone marrow-derived macrophages (BMDMs) and suppressed TNF-α induced-microglia inflammation [11–13]. However, whether PGAM5 affects hepatic steatosis and NASH was unknown. In this study, we employed PGAM5 global-knockout (GKO) mice fed with a high fat high fructose (HFHF) diet and a methionine choline deficient (MCD) diet to investigate the effects of PGAM5 on hepatic steatosis, inflammation and early fibrosis. Besides, we further elucidated the role of PGAM5 on pro-inflammatory response in human and mouse macrophages using in vitro THP-1 and BMDM model, respectively.

## Results

### PGAM5 was up-regulated in NASH

**Fig. 1:**
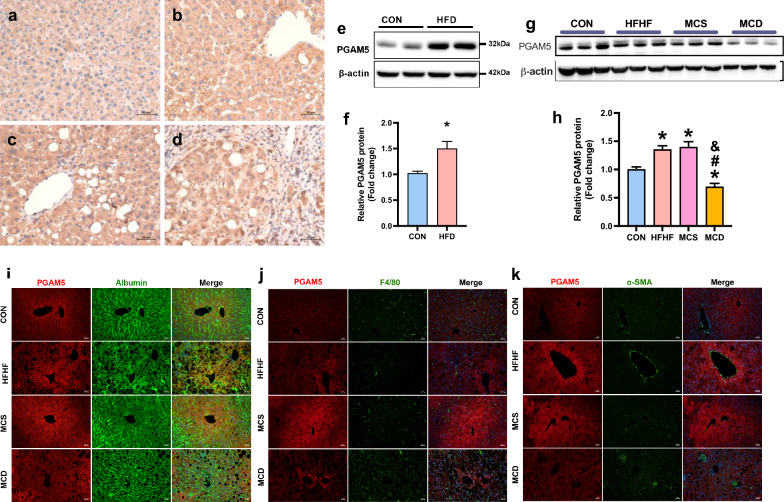
PGAM5 expression is altered in human and mice NASH models. **a**–**d** Identical IHC of PGAM5 in human liver sections with different stages of NASH (**a** healthy donor; **b** steatosis without fibrosis; **c** steatosis with fibrosis stage 1–2; **d** steatosis with fibrosis stage 3–4). Scale bars, 50 μm. **e**, **f** Hepatic PGAM5 protein levels in mice fed with CON and HFD for 20 weeks. *p < 0.05. N = 5. **g**, **h** Hepatic PGAM5 protein levels in wild type mice fed with CON for 20 weeks, HFHF for 12 weeks, MCS and MCD for 6 weeks. *p < 0.05 compared with CON, ^#^p < 0.05 compared with HFHF, ^&^p < 0.05 compared with MCS. N = 5–12. **i**–**k** Representative pictures of hepatic double immunofluorescence staining for PGAM5 (red) and albumin (green)/F4/80 (green) and α-SMA (green) in wildtype fed on CON, HFHF, MCS or MCD diet. Nuclei were stained by DAPI. IHC, immunohistochemistry; CON, control diet; HFD, high fat diet; HFHF, high fat high fructose; MCS, methionine choline supplemented; MCD, methionine choline deficient

In human livers with steatosis, we observed a trend of increase of PGAM5 expression especially around the site of cell ballooning (Fig. 1a, b). In participants with advanced stages of fibrosis, we did not observe any further up-expression of PGAM5 (Fig. 1c, d). In wildtype (WT) C57/BL6 mice, hepatic PGAM5 protein expression was increased by HFD (Fig. 1e, f), HFHF and methionine choline supplemented (MCS) feeding and reduced by MCD feeding (Fig. 1g, h). PGAM5 was primarily expressed in hepatocytes in mice. The PGAM5 expression in hepatocytes around the central vein was up-regulated in WT mice fed on HFHF and MCS diet and reduced in WT mice fed on MCD diet (Fig. 1i). Few PGAM5 expression was detected in Kupfer cells or hepatic satellite cells (HSCs) and the diets did not affect PGAM5 expression in these cells (Fig. 1j–k).

### PGAM5-KO alleviates HFHF-induced obesity and NASH

**Fig. 2:**
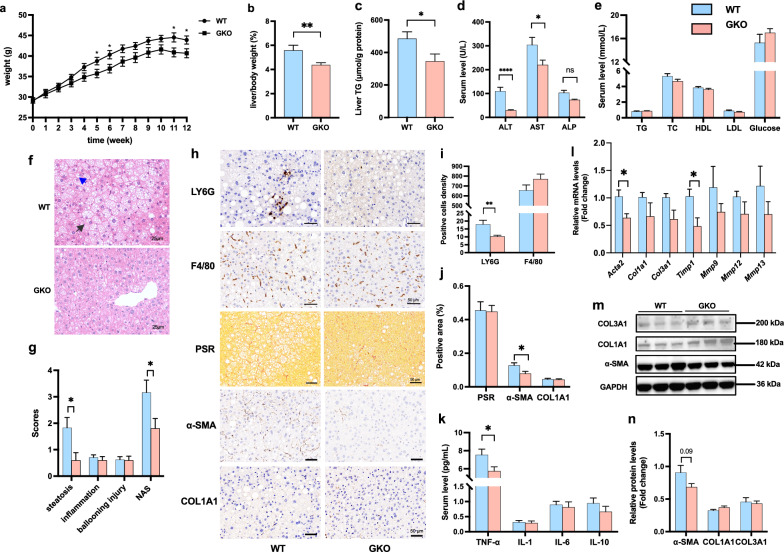
Knockout of PGAM5 improves HFHF-induced obesity and suppresses hepatic inflammation and fibrosis in mice. WT and GKO mice were fed with HFHF for 12 weeks (N = 12–15). **a** Body weight from week 1 to week 12. **b**, **c** Ratio of liver/body weights (**b**) and liver TG content (**c**). **d**, **e** Biochemical measurement of serum ALT, AST and ALP (**d**), lipids and glucose (**e**) in mice. **f–g** Representative H&E staining of liver sections (**f**) and evaluation of NAS (including scores of steatosis, inflammation and ballooning injury) (**g**) in mice. The black arrow indicates scattered inflammation and the blue one indicates ballooning and MDB of hepatocytes. Scale bars, 25 μm. **h**–**j** Representative staining of LY6G and F4/80, PSR staining and IHC stainings for α-SMA and COL1A1 in liver sections and their statistics of positive cells density or positive area (%). Scale bars, 50 μm. **k** Serum levels of pro-inflammatory cytokines (TNF-α, IL-1, IL-6, IL-10) in mice. **l** Hepatic mRNA expressions of pro-fibrotic genes in mice. **m**, **n** Relative hepatic protein levels of α-SMA, COL1A1 and COL3A1 measured byWestern blot. *p < 0.05, **p < 0.01 compared with the WT group. ns, no significance; NAS, NAFLD activity score; ALT, alanine aminotransferase; AST, aspartate aminotransferase; ALP, alkaline phosphatase; TG, triglyceride; TC, total cholesterol; HDL, high density lipoprotein; LDL, low density lipoprotein; MDB, Mallory-Denk body; PSR, picrosirius red; α-SMA (Acta2), α smooth muscle actin; COL, collagen; Mmp, matrix metalloproteases; Timp, tissue inhibitors of Mmps; WT, wild type; GKO, global knockout

PGAM5-GKO mice had significantly decreased weight gain, liver/body weight ratio, and liver triglyceride (TG) compared with WT mice fed with HFHF diet (Fig. 2a–c). Knockout of PGAM5 decreased serum ALT and AST levels (Fig. 2d) without affecting serum lipids, e.g. TG, total cholesterol (TC), high density lipoprotein (HDL) and low density lipoprotein (LDL) (Fig. 2e). Knockout of PGAM5 markedly improved hepatic NAS score primarily by reducing hepatic steatosis (Fig. 2f, g).

GKO mice had significantly reduced hepatic neutrophil and similar macrophage infiltration and reduced α smooth muscle actin (α-SMA) staining with similar picrosirius red (PSR) and Collagen1A1 (COL1A1) staining compared with WT (Fig. 2h–j). Compared with WT group, serum TNF-α levels were significantly decreased despite IL-1, IL-6 and IL-10 were unchanged in GKO mice (Fi.2 k). We also found a significant reduction in α-SMA (Acta2) mRNA expression and a borderline decrease in α-SMA protein level in the liver (Fig. 2l–n). Other fibrotic genes were unchanged except for a decline in Timp1 mRNA expressions in GKO mice (Fig. 2l).

### PGAM5-KO upregulated antioxidative genes in HFHF-fed mice

**Fig. 3:**
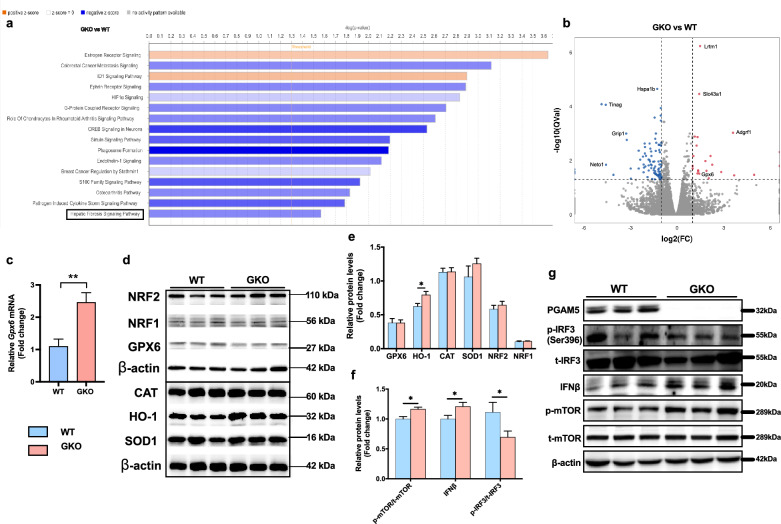
Knockout of PGAM5 promotes expressions of hepatic antioxidative genes and mTOR activation in mice fed with HFHF. **a** IPA analysis was carried out and the top canonical pathways enriched in liver tissues in GKO mice compared with WT were shown. **b** The differentially expressed genes analyzed by volcano plot in GKO v.s WT mice. Red and blue dots represent up-regulated and down-regulated genes, respectively (N = 5). **c** Relative hepatic mRNA expressions of Gpx6 verified by real-time PCR. **d**, **e** Relative hepatic protein levels of antioxidative genes (GPX6, HO-1, CAT, SOD1, NRF2 and NRF1) measured by Western blot. **f**, **g** Relative hepatic protein levels of PGAM5 and known PGAM5 regulated pathways including IFNβ, p-IRF3/t-IRF3 and p-mTOR/t-mTOR measured by Western blot. N = 12–15; *p < 0.05, **p < 0.01 compared with the WT group. IPA, ingenuity pathway analysis; GPX6, glutathione peroxidase 6; HO-1, heme oxygenase-1; CAT, catalase; SOD1, superoxide dismutase 1; NRF2, nuclear factor erythroid 2- related factors; NRF1, nuclear respiratory factor 1; IFNβ, interferon β; IRF3: interferon regulatory factor 3; mTOR, mammalian target of rapamycin; WT, wild type; GKO, global knockout

To further elucidate the transcriptional changes induced by PGAM5, we performed RNA-seq on the liver tissues of HFHF-fed mice. The ingenuity pathway analysis (IPA) suggested an alteration on many canonical pathways including liver fibrosis pathway (Fig. 3a). A total of 112 genes were differentially expressed between WT and GKO mice, of which 23 genes were upregulated and 89 were downregulated (Padj < 0.05; Fig. 3b). Among the differential expressed genes, we verified by real-time PCR that glutathione peroxidase-6 (GPX6), which is an antioxidative gene, was significantly upregulated in GKO mice (Fig. 3c). We expanded our investigation on other antioxidative genes and results showed that protein levels of heme oxygenase-1 (HO-1) were significantly increased in GKO mice (Fig. 3d, e). Furthermore, we examined other known PGAM5-regulated pathways and found that PGAM5-GKO stimulated phosphorylation of mammalian target of rapamycin (mTOR), reduced the phosphorylation of interferon regulatory factor 3 (IRF3), and increased interferon β (IFNβ) protein levels in the livers of GKO mice (Fig. 3f, g).

### PGAM5-KO increased pro-inflammatory response and decreased antioxidative genes in MCS-fed mice

**Fig. 4:**
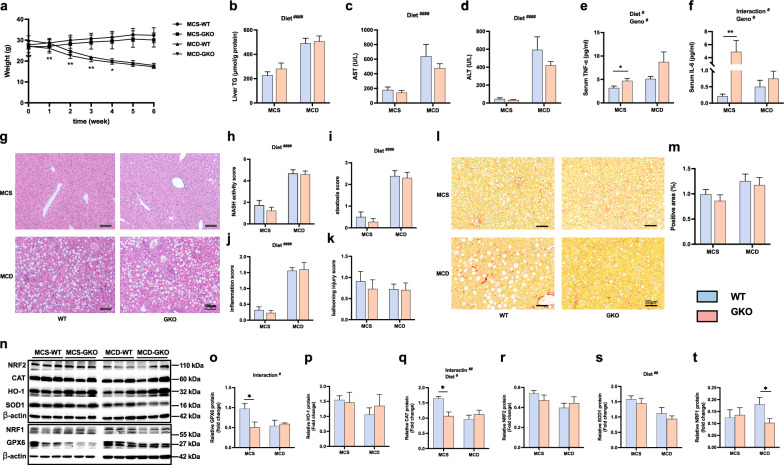
Knockout of PGAM5 shows no protective effect in mice fed with MCS or MCD diet. **a–f** Body weight (**a**), liver TG (**b**), serum AST (**c**) and ALT (**d**), serum levels of TNF-α (**e**) and IL-6 (**f**) were examined in mice fed with MCS or MCD diet for six weeks. **g–k** Representative H&E staining of liver sections and evaluation of NAS (including scores of steatosis, inflammation and ballooning injury). Scale bars, 100 μm. **l**, **m** Representative PSR staining of fibrosis in liver sections and its statistics of positive area (%). Scale bars, 50 μm **n**, **t** Relative hepatic protein levels of antioxidative genes including GPX6 (**o**), HO-1 (**p**), CAT (**q**), NRF2 (**r**), SOD1 (**s**) and NRF1 (**t**) measured by Western blot. N = 9–11; *p < 0.05, **p < 0.01, statistical differences between WT and GKO group. ^#^p < 0.05, ^##^p < 0.01, ^####^p < 0.0001, statistical differences between diets, genotypes or their interactions. TG, triglyceride; ALT, alanine aminotransferase; AST, aspartate aminotransferase; GPX6, glutathione peroxidase 6; HO-1, heme oxygenase-1; CAT, catalase; SOD1, superoxide dismutase 1; NRF2, nuclear factor erythroid 2- related factors; NRF1, nuclear respiratory factor 1; MCS, methionine choline supplemented; MCD, methionine choline deficient; WT, wild type; GKO, global knockout

We also used another NASH model to study the role of PGAM5 deletion. MCD diet resulted in significantly decreased body and liver weight compared to MCS diet-fed mice, and in MCD-fed mice, knockout of PGAM5 significantly decreased body weight compared with its WT control at the first 4 weeks (Fig. 4a). However, no significant difference was found between WT and GKO mice in terms of liver TG and serum AST and ALT levels (Fig. 4b–d). GKO mice fed with MCS had significantly increased serum TNF-α and IL-6 compared to their WT control, while no significant difference was observed in mice fed with MCD diet (Fig. 4 e–f). MCD-fed mice had significantly increased hepatic steatosis and infiltration of inflammatory cells quantified by histology, however, the genotype difference was neglectable (Fig. 4g–k). GKO mice had similar hepatic fibrosis with WT mice on either diet (Fig. 4l, m). We also examined the expression of antioxidative genes in MCS/MCD-fed mice and found that PGAM5 deletion significantly decreased protein levels of GPX6 and catalase (CAT) in MCS-fed mice rather than in MCD-fed mice (Fig. 4n–t).

### PGAM5-knockdown supressed antioxidative genes in vitro

**Fig. 5:**
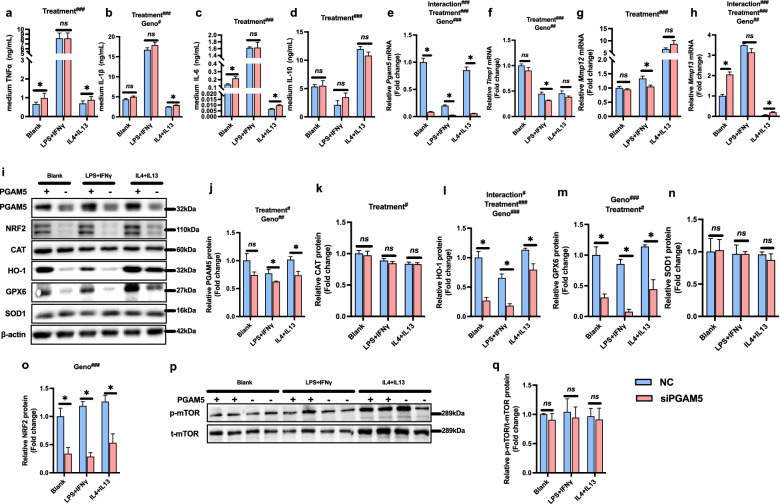
PGAM5 silencing stimulated pro-inflammatory cytokine excretion and down-regulated antioxidative proteins in macrophages. Differentiated THP-1 cells were stimulated with LPS + IFNγ or IL-4 + IL-13 and treated with NC or PGAM5-siRNA for 24–48 h. **a**–**d** Levels of TNF-α (**a**), IL-1β (**b**), IL-6 (**c**) and IL-10 (**d**) secreted in cultural medium. **e**–**h** Relative mRNA expressions of PGAM5 (**e**) and pro-fibrotic genes including Timp1 (**f**), Mmp12 (**g**) and Mmp13 (**h**) measured by Real-time PCR. **i**–**o** Relative protein levels of PGAM5 (**j**) and antioxidative proteins including CAT (**k**), HO-1 (**l**), GPX6 (**m**), SOD1 (**n**) and NRF2 (**o**) measured by Western blot. **p**, **q** Relative protein levels of p-mTOR/t-mTOR measured by Western blot. *p < 0.05 between NC and siPGAM5 group. ^#^p < 0.05, ^##^p < 0.01, ^###^p < 0.001, statistical differences between treatment, genotypes or their interactions. ns, no significance; LPS, lipopolysaccharide; IFNγ, interferon γ; Mmp, matrix metalloproteases; Timp, tissue inhibitors of Mmps; GPX6, glutathione peroxidase 6; HO-1, heme oxygenase-1; CAT, catalase; SOD1, superoxide dismutase 1; NRF2, nuclear factor erythroid 2- related factors; mTOR, mammalian target of rapamycin; NC, negative control; siPGAM5, PGAM5-knockdown

We further investigated the role of PGAM5 silencing in NASH by studying critical human liver cells, including macrophages, hepatocytes, and HSCs in vitro. In human differentiated THP-1 cells (a human leukemia monocytic cell line), knockdown of PGAM5 stimulated TNFα and IL-6 secretion in naïve macropahges and increased TNFα, IL-6 and IL-1β in macropahges treated with IL-4 and IL-13 (M2 macrophage). However, we did not observe any difference in pro-inflamamtory cytokines in LPS and IFN $\gamma$ treated macrophage (M1 macrophage) (Fig. 5 a-d). The mRNA expressions of Timp1 and Mmp12 declined in M1 macrophages and Mmp13 increased in M2 macrophages (Fig. 5f–h). PGAM5-knockdown reduced antioxidative proteins including HO-1, GPX6 and NRF2 in all macrophage subtypes (Fig. 5i–o), but did not affect mTOR phosphorylation (Fig. 5p, q). Furthermore, we tested BMDMs derived from WT and GKO mice, and observed enhanced mRNA expression of pro-inflammatory cytokines (TNFα) and reduced protein levels of GPX6 and CAT (Addtional file : Fig. S1).

**Fig. 6:**
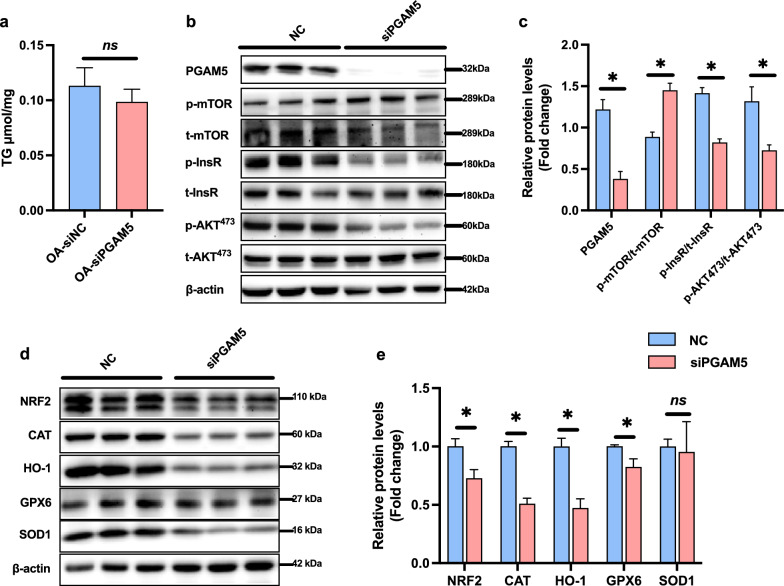
PGAM5 silencing suppressed antioxidative genes in HepG2 cells. HepG2 cells were stimulated with oleic acid (OA) and palmitic acid (PA) and treated with NC or PGAM5-siRNA for 24–48 h. **a** TG levels measured in HepG2 cells. **b**, **c** Relative protein levels of p-mTOR/t-mTOR, p-InsR/t-InsR and p-AKT/t-AKT measured by Western blot. **d**, **e** Relative protein levels of antioxidative proteins including NRF2, CAT, HO-1, GPX6 and SOD1 measured by Western blot. *p < 0.05, statistical differences between NC and siPGAM5 group. ns, no significance; mTOR, mammalian target of rapamycin; InsR, insulin receptor; AKT, protein kinase B; NRF2, nuclear factor erythroid 2- related factors; CAT, catalase; HO-1, heme oxygenase-1; GPX6, glutathione peroxidase 6; SOD1, superoxide dismutase 1; NC, negative control; siPGAM5, PGAM5-knockdown

In human HepG2 cells, PGAM5-knockdown had no effect on fat accumulation in fatty acid treated HepG2 cells (Fig. 6a), but reduced insulin sensitivity (Fig. 6b, c) as well as antioxidative genes including NFR2, CAT, HO-1 and GPX6 (Fig. 6d, e). In TGFβ-stimulated LX-2 cells, PGAM5-knockdown increased mRNA and protein expressions of α-SMA and Col and suppressed mRNA expressions of antioxidative genes including Nrf2, Ho-1, Cat and superoxide dismutase 1 (Sod1) (Additional file 1: Fig. S2).

Thus, PGAM5 deficiency in hepatocyte 'pheno-copied' MCS-model rather than the HFHF-model. Since the systemic obesity and fat accumulation were the primary distinctions between these two animal models, we employed PGAM5 silencing in adipocytes—differentiated 3T3L1 cells. And PGAM5-knockdown resulted in reduced TG accumulation, improved insulin sensitivity, enhanced phosphorylation of mTOR and increased expressions of antioxidative genes such as NRF2, CAT, HO-1 and GPX6 (Additional file 1: Fig. S3).

## Discussion

Our study demonstrated that PGAM5-KO protected mice from HFHF-diet induced NASH in terms of reduced steatosis, liver injury, inflammation and pro-fibrotic genes. However, PGAM5-KO stimulated pro-inflammatory response in mice fed with MCS and had no effect in mice fed with MCD diet. PGAM5 deficiency increased secretions of pro-inflammatory cytokines in human differentiated macrophages (THP-1 cell line) and mouse BMDMs, reduced insulin sensitivity in HepG2 cells and stimulated activation of LX2 cells. PGAM5-KO was found to increase mTOR phosphorylation in certain models, while the expression pattern of antioxidative genes was inversely related to the changes in inflammatory markers across all models.

The major finding of our study was the differential effects of PGAM5 on two NASH models: PGAM5-KO relieved NASH in the HFHF diet model but had no effect in MCD induced NASH. HFHF and MCD are both common diets inducing NASH, but their mechanisms are totally different. In our study, HFHF diet had stronger fat-accumulating effects in the liver, while MCD diet showed stronger pro-inflammatory and pro-fibrotic effects, which was similar to other studies [14]. The HFHF-induced model recapitulated the natural history of human NAFLD because hepatic steatosis was largely owing to excessive energy intake, whereas MCD model had the disadvantage of drastic weight loss due to energy depletion. In fact, these two diets regulated hepatic PGAM5 expression in opposite ways (Fig. 1), suggesting possible differential roles of PGAM5 on these two models. A previous study already demonstrated that PGAM5-KO may alleviate obesity-related metabolic disorders (e.g. glucose intolerance) [9]. In this study, we found that knockout of PGAM5 could improve liver inflammation induced by HFHF diet, which is a key process of NASH. Previous studies suggested that PGAM5 deficiency protected mice from ConA-induced hepatocellular death and thus showed a robust effect in reducing serum aminotransferase [10]. The resolving of hepatic injury in conA-induced acute hepatitis model was largely resulting from the regulative effect of PGAM5 on programmed cell death [7, 10, 15]. Our data suggested that the role of PGAM5 on NASH was only prominent with the presence of obesity since PGAM5 deletion only alleviated NASH in the HFHF model with excessive energy intake and systemic fat accumulation. This aligns with the phenomenon that certain mitochondrial proteins may respond to overabundance or scarcity of nutrients in unique or even opposing manners, in order to maintain a balance between energy expenditure and supply [16].

We tried to illucidate the cellular mechanism by supressing PGAM5 expression in all major hepatic cells involved in NASH. None of the three hepatic cell types recapitulated the ‘protective’ phenotype of PGAM5-KO in HFHF model. With PGAM5 knockdown, insulin resistance was increased in HepG2 cells, pro-inflammatory cytokines secretion was enhanced in THP-1 and BMDM cells, and pro-fibrotic genes were up-regulated in LX2 cells. The phenotype in macrophages recapitulated the profile of inflammatory markers in MCS mouse model, in which serum TNF-α and IL-6 were increased in PGAM5-KO group. A recent study reported that knockout of PGAM5 showed a trend of increase in mRNA expressions of IL-6 in BMDMs treated with LPS [12]. However, the effects of PGAM5 on BMDM may be complicated. Knockout of PGAM5 significantly decreased expression and secretion of IL-1 in BMDMs treated with LPS plus inflammasome agonists [12]. Besides, other studies showed that PGAM5 is needed for the activation of NKT cells or inflammatory factors [11, 17]. These suggest the regulation of macrophage and inflammatory cells by PGAM5 varied among different models. The in vivo effects of PGAM5-KO in the HFHF model were only replicated in 3T3L1 cells, where PGAM5 silencing led to a decrease in TG accumulation and improved insulin sensitivity. This may be due to roles of PGAM5 plays in adipose tissue, as its reduction could result in decreased adiposity, leading to reduced systemic inflammation and subsequent improvement in NASH.

We tried to dissect the role of PGAM5 by testing known intrinsic pathways associated with PGAM5 and explore other possible pathways by RNAseq. It is reported that PGAM5 deletion could result in activation of mTORs and IRF3/IFNβ pathyways which were involved in cell senescence process [18]. However, in our HFHF model, we did not observe an upregulation of the IRF3/IFNβ pathway. Although IFNβ expression was increased in PGAM5-KO mice fed with HFHF, IRF3 phosphorylation was reduced. The IRF3/IFNβ pathway plays a role in innate immune response and hepatic inflammation [19]. Studies showed that IFNβ may limit HSC activation [20] and reduction in IRF3 activation may lead to hepatic insulin resistance and steatosis [21], which contradicted with the phenotype of our HFHF model. It is possible that IFNβ was regulated by other transcriptional factors, e.g. NFkB, rather than IRF3 [19]. Therefore, we speculate that IRF3 pathway may be not the key component involved in the relationship between PGAM5 and NASH, so we did not further test this pathway in our cell models.

mTOR is a key nutrient-sensing molecule that regulates various signaling pathways related to 'growth' during energy oversupply [22]. It is repoted that dietary restriction, which mimics MCD treatment, can reduce mTOR activity [23], and this suggests that mTOR could potentially mediate the effect of PGAM5. We found that PGAM5 silencing increased mTOR phosphorylation in livers of HFHF-fed mice, and in vitro HepG2 and 3T3L1 cells, which are all constructed nutrient-oversupply models. In addtion, mTOR phosphorylation was unchanged in THP-1 cells, which represent balanced nutrition state. While the upregulation of mTOR by PGAM5 silencing may be energy-dependent, it cannot fully explain the opposite effects on insulin sensitivity in HepG2 and 3T3L1 cells, despite similar increases in mTOR activity. Other mechanisms beyond energy-dependent responses may be involved in the regulation of PGAM5-KO in NASH.

**Fig. 7:**
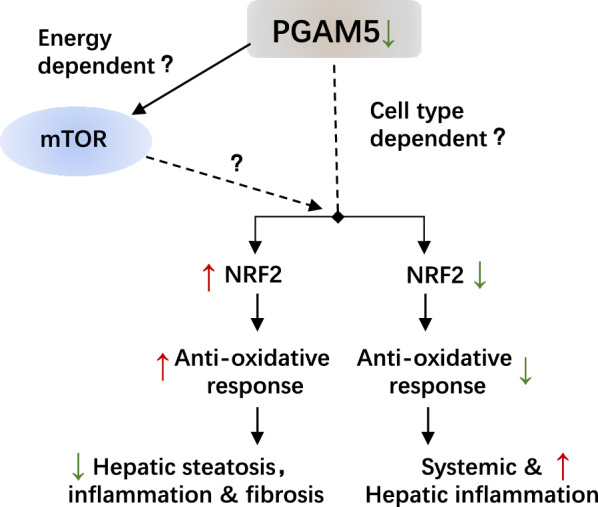
Proposed model for PGAM5 suppression in NASH. PGAM5 played differential roles in different NASH models. We found the expression of antioxidative genes were differentially regulated in mice fed with HFHF, MCS or MCD diets, and in a variety of in vitro models, which further triggered different consequences of obesity, hepatic steatosis, inflammation and fibrosis. The reason that what triggers this change was still unclear. One possibility that this difference was energy dependent and the regulations of PGAM5 on energy sensing gene mTOR may be involved. Another possibility that the differential role of PGAM5 in antioxidative response may be energy dependent or cell type dependent

By RNA sequencing, we confirmed that GPX6 was increased in PGAM5-KO mice fed with HFHF and we found that expressions of GPX6 and other antioxidative genes showed an opposite pattern with hepatic inflammation in all models. This highlights the role of PGAM5 on oxidative stress and inflammation. Likewise, another study showed that HO-1 protected the liver against ischemia/reperfusion via PGAM5 dependent pathway, suggesting the interplay between PGAM5 and antioxidative proteins [24]. Besides, it is reported that apoptosis inducing factor (AIF), which has a role in maintaining mitochondrial energy homeostasis, binds to PGAM5 and can reduce the ability of PGAM5 to control antioxidant responses [25], which indicates that the differential effects of PGAM5-KO on the antioxidant process and inflammation may also rely on the different energy status of the models, possibly through the regulation of NRF2. Similarly, previous study suggested that the role of PGAM5-KO on NRF2 was age dependent [18]. Alghouth NRF2 was not regulated in our in vivo models, it was significantly changed in our in vitro models and showed opposing trend with pro-inflammatory response. As a result, we proposed that the possible mechanism of PGAM5 deletion on NASH may result from the differential effects on antioxidative genes, leading to identical inflammatory and metabolic phenotypes (Fig. 7). However, the energy-dependent and/or cell-type-dependent manner that triggers the different effect of PGAM5 on NRF2 and the subsequent inflammatory response requires further validation.

To our knowledge, this is the first study to investigate the role of PGAM5 in chronic metabolic liver disease using global knockout mouse models. Also, our study highlighted the possibility of PGAM5 inhibitors as a novel therapeutic target for obesity and NASH. However, we have the following limitations: (1) although we used two models as NASH, both were not ideal. HFHF recapitulated the human natural process, but the inflammation and fibrosis were mild in the liver. MCD induced advanced inflammation and fibrosis in the liver, but it represented nutrient undersupply status and might affect the effects of PGAM5 deletion; (2) we cannot establish the causal relationship between PGAM5, antioxidative proteins and hepatic inflammation since we cannot manipulate antioxidative proteins in our models; and (3) we did not find an ideal cell model to recapitulate in vivo status due to the difficulty of manipulating energy supply in inflammatory cells.

## Conclusions

In conclusion, there were distinct effects of PGAM5 deletion on steatosis, inflammation and fibrosis in NASH models induced by HFHF and MCD (MCS as control) diets. The distinct effects on inflammatory response in NASH may be owing to the different effects of PGAM5-KO on antioxidative pathways in an energy dependent and/or cell-type dependent manner. Our study supports PGAM5 as a novel therapeutic target for obesity and NASH.

## Methods

### Human samples

In this study, four liver tissue samples from three biopsy-proven steatosis or NASH patients and one healthy donor were obtained from the Department of Pathology at Peking University People’s Hospital. Staging of NASH were conducted and confirmed by experienced pathologists. Informed consent forms were obtained from all participants.

### Animals

Wildtype (WT) C57BL/6J mice were fed with control diet (Research Diets D12450J) and HFD (Research Diets D12492) for 20 weeks. Livers were harvested to detect the changes of PGAM5 protein. PGAM5-GKO C57BL/6J mice were constructed by CRISPR/Cas 9 at Cyagen company (China). Mice were housed in the specific pathogen-free (SPF) facility. PGAM5-GKO mice and their WT littermates were fed with high-fat diet (60% fat) and high-fructose (10%) drinking water (TROPHIC, Nantong, China) for 12 weeks or fed with MCD diet (MCS diet as control) (TROPHIC, Nantong, China) for 6 weeks to induce NAFLD/NASH. Male mice aged 8–14 weeks were used. And mice were fasted for 6 h before they were euthanized. N = 9–15/group. All of the animal experiments were approved by the Animal Research Committee of the Peking University People’s Hospital.

### Blood tests

Blood biochemistry including alanine aminotransferase (ALT), aspartate aminotransferase (AST), high-density lipoprotein (HDL), low-density lipoprotein (LDL), triglyceride (TG), total cholesterol (TC), glucose and alkaline phosphatase (ALP) were measured by Roche analyzer (Roche, Basle, Switzerland). Serum pro-inflammatory cytokines including IL-1, IL-6, IL-10 and TNF-α were measured by Cytometric Bead Array (CBA, BD, New Jersey, USA) according to the manufacturer’s protocol.

### Liver histopathology, immunohistochemistry (IHC) and immunofluorescence (IF)

Livers were perfused with PBS through the portal vein before they were dissected. Isolated livers were fixed in 4% paraformaldehyde to make paraffin-embedded blocks and each section was stained with hematoxylin and eosin (H&E). Two investigators were blinded and evaluated the sections using NAFLD activity score (NAS) in 200× magnification. Antibodies of LY6G and F4/80 (Invitrogen, Waltham, USA) were used to detect hepatic neutrophils and macrophages, respectively. Liver sections were stained with picrosirius red (PSR, Servicebio, Wuhan, China) to examine fibrosis. IHC including α-SMA (Abcam, Cambridge, UK) and COL1A1 (CST, Danvers, USA) for mouse liver sections and PGAM5 (Abcam, Cambridge, UK) for human liver sections with different stages of NASH were also conducted. Double IF used PGAM5 (Proteintech, Wuhan, China) with secondary antibody attached with Cyanine Dye 3 (CY3) and albumin (Abcam, Cambridge, UK), F4/80 (Proteintech, Wuhan, China) or α-SMA (Abcam, Cambridge, UK) with secondary antibody attached with fluorescein isothiocyanate (FITC). Nuclei were stained by 4′,6-diamidino-2-phenylindole (DAPI). For density quantification, 20 to 30 high-power fields per section were randomly selected for each slide by an assessor blind to genotype and were analyzed using Halo (Indica labs, New Mexico, USA) for positively stained pixels and normalized to the total number of pixels of this section.

### Cell culture

Human monocytic cell line (THP-1) and human hepatocarcinoma (HepG2) cell line were obtained from the American Type Culture Collection (ATCC). HepG2 were cultured in low glucose complete DMEM medium (1.0 g/L glucose, 10% FBS and 1% penicillin/streptomycin (P/S)), and THP-1 were cultured in RPMI 1640 (Gibco, USA) containing 10% FBS and 1% P/S at 37 °C with 5% CO2.

#### Treatment of THP-1

THP-1 monocytes were cultured in 12-well plates treated with 100 nmol/L phorbol 12-myristate 13-acetate (PMA; MedChemExpress, USA) for 24 h to transform into adherent M0 macrophages. To obtain M1-polarized macrophages, THP-1 cells were treated with 100 ng/mL lipopolysaccharide (LPS; Merck, USA) plus 20 ng/mL interferon γ (IFNγ, MedChemExpress, USA). To induce M2-polarized macrophages, THP-1 cells were treated with 20 ng/mL IL-4 (PEPROTECH, USA) plus 20 ng/mL IL-13 (MedChemExpress, USA). During polarization, THP-1 cells were transfected with 50 nmol/L PGAM5-siRNA (siPGAM5) or negative control (NC) using jetPrime (Polyplus, France) according to the manufacturer’s instructions. RNA was harvested after 24 h treatment and cultural medium and proteins were obtained after 48 h treatment.

#### Treatment of HepG2

To establish an in vitro model of hepatic steatosis, HepG2 cells were cultured in complete medium supplemented with 200 μM oleic acid (OA; Sigma-Aldrich, USA) and 100 μM palmitic acid (PA; Sigma-Aldrich, USA), and transfected with 50 nmol/L siPGAM5 or NC. RNA and protein were obtained after 24 h or 48–72 h treatement, respectively.

The sequences of mouse siPGAM5 were 5′-GGAGAAGACGAGUUGACAUTT-3′ (forward) and 5′-AUGUCAACUCGUCUUCUCCTT-3′ (reverse). The sequences of human siPGAM5 were 5′- CACUGUCUCUGAUCAACGUTT-3′ (forward) and 5′- ACGUUGAUCAGAGACAGUGTT-3′ (reverse). A scrambled siRNA was used as NC. All siRNAs were designed by Genepharma (Jiangsu, China).

### Western blot (WB)

Proteins were isolated from liver tissues or cells using RIPA lysis buffer (Invitrogen) supplemented with protease inhibitors (Thermo Scientific, Waltham, USA). Proteins were separated using tris–glycine gel (APPLYGEN, Beijing, China) and transferred from the gel to a PVDF transfer membrane (Merck Millipore, Boston, USA). After blocking with 10% skim milk (BD), membranes were probed with the following primary antibodies (details can be found in Additional file 1: Table S1): PGAM5, α-SMA, COL1A1, COL3A1, GPX6, CAT, HO-1, SOD1, nuclear respiratory factor 1 (NRF1), NRF2, phosphor-mTOR (p-mTOR), mTOR, phosphor-insulin receptor β (p-InsRβ), InsRβ, protein kinase B (PKB/AKT), phospho-AKT (p-AKT), glyceraldehyde-3-phosphate dehydrogenase (GAPDH) and β-actin. Horseradish peroxidase (HRP)-linked anti-rabbit, anti-rat and anti-mouse were used as secondary antibodies. The protein bands were visualized by enhanced chemiluminescence detection reagents (APPLYGEN). Pixels were quantified in image J software (NIH, USA). Protein levels were corrected for β-actin or GAPDH levels.

### RNA preparation and real-time PCR analysis

Total RNA was extracted using RNA extraction kit (TaKaRa, Shiga, Japan). cDNA was synthesized using RT reagent kit (TaKaRa) and analyzed by real-time PCR using SYBR Green Master Mix (Applied Biosystems, Waltham, USA). Experimental values were normalized to expressions of the housekeeping gene β-actin. Sequences of tested genes were shown in Additional file 1: Table S2.

### RNA sequencing and data analysis

RNA extraction, RNA-seq and data analysis were performed at Shanghai Biotechnology Corporation (Shanghai, China). Total RNA was extracted using RNAiso Plus Total RNA extraction reagent (TaKaRa) following the manufacturer’ s instructions and checked for a RIN number to inspect RNA integrity by an Agilent 2100 Bioanalyzer (Agilent technologies, USA). Qualified total RNA was further purified by RNAClean XP Kit (Beckman) and RNase-Free DNase Set (QIAGEN, Germany). Purified libraries were quantified by Qubit® 2.0 Fluorometer (Life Technologies, USA) and validated by Agilent 2100 bioanalyzer (Agilent Technologies, USA) to confirm the insert size and calculate the mole concentration. A cluster was generated by cBot with the library diluted to 10 pM and then sequenced on the Illumina HiSeq Xten (Illumina, USA). Differentially expressed genes were identified using edgeR. The p-value significance threshold in multiple tests was set by the false discovery rate (FDR). The fold changes were also estimated according to the FPKM in each sample. The differentially expressed genes were selected using the following filter criteria: FDR ≤ 0.05 and fold-change ≥ 2. These differentially expressed genes, containing gene identifiers and corresponding expression values, were uploaded into the ingenuity pathway analysis (IPA) software (Qiagen, German). The 'core analysis' function included in the software was used to interpret the differentially expressed data, which included biological processes, canonical pathways, upstream transcriptional regulators, and gene networks. Each gene identifier was mapped to its corresponding gene object in the Ingenuity Pathway Knowledge Base (IPKB). Volcano plot were made in R package.

#### ELISA

Cell culture supernatants were collected after 48 h of culture and IL-1β, IL-6, IL-10 and TNF-α were measured by ELISA kit (Dakewe, Guangdong, China) following manufacturer’s instructions. Detailed cataloge information were presented in Additional file 1: Table S3.

#### TG assessment

TG concentrations in cell and tissue lysates were determined using commercial assay kits under the guidance of the manufacturer’s instructions (Nanjingjiancheng, China). The results were normalized by total protein content measured using a BCA Protein Assay Kit (Thermo Scientific, USA).

### Statistical analysis

Data are expressed as the mean ± SEM. For HFHF experiment, two groups were compared using unpaired two-tailed Student’s *t*-test. For other experiments, multiple groups were compared using two-way analysis of variance (two-way ANOVA) followed by the Tukey post hoc analysis. p < 0.05 was considered statistical significance. All statistical data were calculated with GraphPad Prism 9 (San Diego, CA, USA).

### Supplementary Information

**Additional file 1: Method S1.** **Table S1.** Sources of antibodies used in Western blot. **Table S2.** Sequences of genes detected in real-time PCR. **Table S3. **Sources of ELISA kit used in cytokines assay. **Figure S1.** Knockout of PGAM5 increased mRNA expressions of pro-inflammatory genes and decreased protein levels of antioxidative genes in BMDMs. a-d: Relative mRNA expressions of pro-inflammatory cytokines including IL-1 (a) , IL-6 (b), TNF-α (c) and IL-10 (d) measured by real-time PCR  in BMDMs. e-j: Relative protein levels of CAT (f), HO-1 (g), NRF2 (h), GPX6 (i) and SOD1 (j) measured by Western blot. ^*^p<0.05, ^**^p<0.01, ^***^p<0.001, ^****^p<0.0001, statistical differences between WT and KO group. ^#^p<0.05, ^##^p<0.01, ^###^p<0.001, ^####^p<0.0001, statistical differences between treatments, genotypes or their interactions. ns, no significance; LPS, lipopolysaccharide; GPX6, glutathione peroxidase 6; HO-1, heme oxygenase-1; CAT, catalase; SOD1, superoxide dismutase 1; NRF2, nuclear factor erythroid 2- related factors; WT, wild type; KO, knockout. **Figure S2.** Knockdown of PGAM5 increased expressions of pro-fibrotic and decreased mRNA expressions of antioxidative genes in LX2 cells. After treated with TGF for 24h, a-g: Relative mRNA expressions of pro-fibrotic genes including Acta2 (a) , Col1a1 (b), Col3a1 (c), Timp1 (d) Mmp9 (e) and Mmp13 (f), and mRNA expressions of PGAM5 (g) were shown. h-k: Relative mRNA expressions of antioxidative genes including Ho-1 (h) , Nrf2 (i), Sod1 (j) and Cat (k) were shown. l-r: Relative protein levels of COL3A1 (m), MMP13 (n), α-SMA (o), MMP9 (p), COL1A1 (q) and PGAM5 (r) were detected in LX2 cells. *p < 0.05, **p < 0.01, ***p < 0.001, ****p < 0.0001, statistical differences between NC and PGAM5^-/-^ group. ^#^p<0.05, ^##^p<0.01, ^###^p<0.001, ^####^p<0.0001, statistical differences between treatments, genotypes or their interactions. COL, collagen; Mmp, matrix metalloproteases; Timp1, tissue inhibitors of Mmps; Ho-1, heme oxygenase-1; Nrf2, nuclear factor erythroid 2- related factors; Sod1, superoxide dismutase 1; Cat, catalase; NC, negative control; PGAM5^-/-^, PGAM5-knockdown. **Figure S3.** Knockdown of PGAM5 reduced TG accumulation and up-regulated antioxidative genes in 3T3L1 cells. a: TG levels measured in adipocytes. b-g: Relative protein levels of p-mTOR/t-mTOR (b-c), proteins of insulin transduction pathway including p-InsR/t-InsR and p-AKT/t-AKT (d-e) and antioxidative proteins including NRF2, CAT, HO-1 and GPX6 (f-g) were measured in 3T3L1 cells. ^*^p < 0.05, statistical differences between NC and siPGAM5 group. mTOR1, mammalian target of rapamycin complex 1; InsR, insulin receptor β; AKT, protein kinase B; NRF2, nuclear factor erythroid 2- related factors; CAT, catalase; HO-1, heme oxygenase-1; GPX6, glutathione peroxidase 6; NC, negative control; siPGAM5, PGAM5 knockdown. **Figure S4.** Original blots for Figs. 1–4. **Figure S5.** Original blots for Figs. 5, 6.

## Data Availability

The datasets used and/or analysed during the current study are available from the corresponding author on reasonable request.

## Abbreviations

PGAM5: Phosphoglycerate mutase 5
NAFLD: Nonalcoholic fatty liver disease
NASH: Nonalcoholic steatohepatitis
GKO: Global knockout
WT: Wild type
HFHF: High fat high fructose diet
MCD: Methionine choline-deficient
MCS: Methionine choline supplemented
BMDM: Bone marrow-derived macrophages
LPS: Lipopolysaccharide
α-SMA: α Smooth muscle actin
COL: Collagen
Mmp: Matrix metalloproteinase
Timp1: Tissue inhibitors of Mmps
GPX6: Glutathione peroxidase-6
HO-1: Heme oxygenase-1
CAT: Catalase
SOD1: Superoxide dismutase 1
NRF2: Nuclear factor erythroid 2- related factors
mTOR: Mammalian target of rapamycin
IFNβ: Interferon β
IRF3: Interferon regulatory factor 3

## References

[1] Le MH, Yeo YH, Li X, Li J, Zou B, Wu Y, Ye Q (2021). 2019 global NAFLD prevalence: a systematic review and meta-analysis. Clin Gastroenterol Hepatol.

[2] Chalasani N, Younossi Z, Lavine JE, Charlton M, Cusi K, Rinella M, Harrison SA (2018). The diagnosis and management of nonalcoholic fatty liver disease: practice guidance from the American Association for the Study of Liver Diseases. Hepatology.

[3] Buzzetti E, Pinzani M, Tsochatzis EA (2016). The multiple-hit pathogenesis of non-alcoholic fatty liver disease (NAFLD). Metabolism.

[4] Trautwein C, Friedman SL, Schuppan D, Pinzani M (2015). Hepatic fibrosis: concept to treatment. J Hepatol.

[5] Zhou JE, Sun L, Liu L, Jia Y, Han Y, Shao J, Wang J (2022). Hepatic macrophage targeted siRNA lipid nanoparticles treat non-alcoholic steatohepatitis. J Control Release.

[6] Tilg H, Moschen AR (2010). Evolution of inflammation in nonalcoholic fatty liver disease: the multiple parallel hits hypothesis. Hepatology.

[7] Cheng M, Lin N, Dong D, Ma J, Su J, Sun L (2021). PGAM5: A crucial role in mitochondrial dynamics and programmed cell death. Eur J Cell Biol.

[8] Sugawara S, Kanamaru Y, Sekine S, Maekawa L, Takahashi A, Yamamoto T, Watanabe K (2020). The mitochondrial protein PGAM5 suppresses energy consumption in brown adipocytes by repressing expression of uncoupling protein 1. J Biol Chem.

[9] Sekine S, Yao A, Hattori K, Sugawara S, Naguro I, Koike M, Uchiyama Y (2016). The ablation of mitochondrial protein phosphatase Pgam5 confers resistance against metabolic stress. EBioMedicine.

[10] He GW, Gunther C, Kremer AE, Thonn V, Amann K, Poremba C, Neurath MF (2017). PGAM5-mediated programmed necrosis of hepatocytes drives acute liver injury. Gut.

[11] Kang YJ, Bang BR, Han KH, Hong L, Shim EJ, Ma J, Lerner RA (2015). Regulation of NKT cell-mediated immune responses to tumours and liver inflammation by mitochondrial PGAM5-Drp1 signalling. Nat Commun.

[12] Moriwaki K, Farias Luz N, Balaji S, De Rosa MJ, O'Donnell CL, Gough PJ, Bertin J (2016). The mitochondrial phosphatase PGAM5 is dispensable for necroptosis but promotes inflammasome activation in macrophages. J Immunol.

[13] Wang Y, Bi Y, Xia Z, Shi W, Li B, Li B, Chen L (2018). Butylphthalide ameliorates experimental autoimmune encephalomyelitis by suppressing PGAM5-induced necroptosis and inflammation in microglia. Biochem Biophys Res Commun.

[14] Arao Y, Kawai H, Kamimura K, Kobayashi T, Nakano O, Hayatsu M, Ushiki T (2020). Effect of methionine/choline-deficient diet and high-fat diet-induced steatohepatitis on mitochondrial homeostasis in mice. Biochem Biophys Res Commun.

[15] Wang Z, Jiang H, Chen S, Du F, Wang X (2012). The mitochondrial phosphatase PGAM5 functions at the convergence point of multiple necrotic death pathways. Cell.

[16] Schrepfer E, Scorrano L (2016). Mitofusins, from mitochondria to metabolism. Mol Cell.

[17] Kang TB, Yang SH, Toth B, Kovalenko A, Wallach D (2013). Caspase-8 blocks kinase RIPK3-mediated activation of the NLRP3 inflammasome. Immunity.

[18] Yu B, Ma J, Li J, Wang D, Wang Z, Wang S (2020). Mitochondrial phosphatase PGAM5 modulates cellular senescence by regulating mitochondrial dynamics. Nat Commun.

[19] Zhang C, Liu S, Yang M (2022). The role of interferon regulatory factors in non-alcoholic fatty liver disease and non-alcoholic steatohepatitis. Gastroenterol Insights.

[20] Shimozono R, Nishimura K, Akiyama H, Funamoto S, Izawa A, Sai T, Kunita K (2015). Interferon-β mediates signaling pathways uniquely regulated in hepatic stellate cells and attenuates the progression of hepatic fibrosis in a dietary mouse model. J Interferon Cytokine Res.

[21] Wang X-A, Zhang R, She Z-G, Zhang X-F, Jiang D-S, Wang T, Gao L (2014). Interferon regulatory factor 3 constrains IKKβ/NF-κB signaling to alleviate hepatic steatosis and insulin resistance. Hepatology.

[22] Sabatini DM (2017). Twenty-five years of mTOR: uncovering the link from nutrients to growth. Proc Natl Acad Sci.

[23] Green CL, Lamming DW, Fontana L (2022). Molecular mechanisms of dietary restriction promoting health and longevity. Nat Rev Mol Cell Biol.

[24] Hong JM, Lee SM (2018). Heme oxygenase-1 protects liver against ischemia/reperfusion injury via phosphoglycerate mutase family member 5-mediated mitochondrial quality control. Life Sci.

[25] Lenhausen AM, Wilkinson AS, Lewis EM, Dailey KM, Scott AJ, Khan S, Wilkinson JC (2016). Apoptosis inducing factor binding protein PGAM5 triggers mitophagic cell death that is inhibited by the ubiquitin ligase activity of X-linked inhibitor of apoptosis. Biochemistry.

