# Supplementary material for: Differential effects of PGAM5 knockout on high fat high fructose diet and methionine choline-deficient diet induced non-alcoholic steatohepatitis (NASH) in mice
Source: Cell Biosci. 2023 Aug 21;13:154. doi: 10.1186/s13578-023-01095-3 (PMC10440915; doi:10.1186/s13578-023-01095-3)
Supplement: Supplementary file 1 — Additional file 1: Method S1. Table S1. Sources of antibodies used in Western blot. Table S2. Sequences of genes detected in real-time PCR. Table S3. Sources of ELISA kit used in cytokines assay. Figure S1. Knockout of PGAM5 increased mRNA expressions of pro-inflammatory genes and decreased protein levels of antioxidative genes in BMDMs. a-d: Relative mRNA expressions of pro-inflammatory cytokines including IL-1 (a) , IL-6 (b), TNF-α (c) and IL-10 (d) measured by real-time PCR in BMDMs. e-j: Relative protein levels of CAT (f), HO-1 (g), NRF2 (h), GPX6 (i) and SOD1 (j) measured by Western blot. *p<0.05, **p<0.01, ***p<0.001, ****p<0.0001, statistical differences between WT and KO group. #p<0.05, ##p<0.01, ###p<0.001, ####p<0.0001, statistical differences between treatments, genotypes or their interactions. ns, no significance; LPS, lipopolysaccharide; GPX6, glutathione peroxidase 6; HO-1, heme oxygenase-1; CAT, catalase; SOD1, superoxide dismutase 1; NRF2, nuclear factor erythroid 2- related factors; WT, wild type; KO, knockout. Figure S2. Knockdown of PGAM5 increased expressions of pro-fibrotic and decreased mRNA expressions of antioxidative genes in LX2 cells. After treated with TGF for 24h, a-g: Relative mRNA expressions of pro-fibrotic genes including Acta2 (a) , Col1a1 (b), Col3a1 (c), Timp1 (d) Mmp9 (e) and Mmp13 (f), and mRNA expressions of PGAM5 (g) were shown. h-k: Relative mRNA expressions of antioxidative genes including Ho-1 (h) , Nrf2 (i), Sod1 (j) and Cat (k) were shown. l-r: Relative protein levels of COL3A1 (m), MMP13 (n), α-SMA (o), MMP9 (p), COL1A1 (q) and PGAM5 (r) were detected in LX2 cells. *p < 0.05, **p < 0.01, ***p < 0.001, ****p < 0.0001, statistical differences between NC and PGAM5-/- group. #p<0.05, ##p<0.01, ###p<0.001, ####p<0.0001, statistical differences between treatments, genotypes or their interactions. COL, collagen; Mmp, matrix metalloproteases; Timp1, tissue inhibitors of Mmps; Ho-1, heme oxygenase-1; Nrf2, nuclear factor erythroi [file 13578_2023_1095_MOESM1_ESM.docx]

**Additional file**

**Method S1:**

3T3-L1 cell culture

The murine 3T3L1 preadipocyte cell line was purchased from the American Type Culture Collection (ATCC). 3T3-L1 preadipocytes were cultured in high glucose complete medium (4.5 g/L Debulcos Modified Eagles Medium (DMEM; Gibco, USA), 10% fetal bovine serum (FBS; Gibco, USA) and 1% penicillin/streptomycin (P/S; Gibco, USA)) in incubator at 37 °C with 5 % CO2.

To differentiate into mature adipocytes, 3T3-L1 cells were seeded in 12-well culture plate (Corning, USA) and allowed to grow until fully confluent. Two days after confluence (Day 0), the cells were cultured in differentiation medium (0.5mM 3-isobutyl-1-methylxanthine (IBMX), 1μM dexamethasone and 10μg/mL bovine insulin, Sigma-Aldrich, USA ) for 3 days (Day 3). These cells were then cultured in adipocyte maintenance medium containing DMEM supplemented with 10% FBS, 10μg/mL bovine insulin. The old adipocyte maintenance medium was replaced every 48 h and cells were fully differentiated after 15 days of induction (Chi Oncotarget 2017). To knockdown PGAM5 gene expression, mature 3T3-L1 cells were cultured in complete medium containing 200μM Oleic acid (OA, Sigma-Aldrich, USA ) and 100μM palmitic acid (PA, Sigma-Aldrich, USA) in 12-well plates, and transfected with 50 nmol/L NC or siPGAM5 produced by Genepharma (Jiangsu, China) using jetPrime (Polyplus, France) according to the manufacturer’s instructions.

LX-2 cell culture

LX-2, a well-characterized cell line derived from human HSCs, was used in *in vitro* studies. Cells were cultured in 1640 medium (Gibco, Shanghai, China) supplemented with 10% fetal bovine serum (Gibco, Shanghai, China) and 1% P/S. For transfection, LX-2 cells were plated at 1×10^5^ cells/well in 6-well plates and cultured overnight. Cells were transfected with 50 nmol/L NC or siPGAM5 produced by GenePharma (Jiangsu, China) using Lipofectamine 3000 (Invitrogen, Shanghai, China).

BMDM differentiation and culture

Primary bone marrow derived macrophage (BMDMs) were isolated from the tibiae and femurs of WT and GKO mice. Briefly, bone marrow was washed and collected by sterilized PBS, after centrifugation, the lysis buffer (Servicebio) was added to remove the red blood cells and cells were purified through a 70µm strainer. Resuspended BMDMs were cultured in DMEM containing 20 ng/ml macrophage/monocyte‐colony stimulating factor (M‐CSF, Proteintech, Rosemont, USA) for seven days. The purity of the cultured BMDM was assessed by flow cytometry. Preparations with >95% of the cells expressed F4/80 were valid for further experiment (Tang, Adv Sci (Weinh) 2022). BMDMs were further treated by 100 ng/ml LPS (Sigma, Saint Louis, USA) or 20 ng/ml IL-4 (Proteintech) for 24 h to induce M1-like or M2-like macrophages, respectively. Cells were washed and assessed by a Flow Cytometer (Beckman Coulter, Brea CA, USA). Cells with F4/80^+^CD11b^+^MHCII^+^ were regarded as M1 and F4/80^+^CD11b^+^CD206^+^ were regarded as M2 macrophage.

**Additional tables**

Table S1. Sources of antibodies used in Western blot.

| **Antibodies** | **Manufacturer** |
| --- | --- |
| PGAM5 | Abcam, Cambridge, UK |
| α-SMA | Abcam, Cambridge, UK |
| COL1A1 | CST, Danvers, USA |
| COL3A1 | CST, Danvers, USA |
| p-InsRβ | CST, Danvers, USA |
| InsRβ | CST, Danvers, USA |
| p-AKT | CST, Danvers, USA |
| AKT | CST, Danvers, USA |
| p-IRF3(Ser396) | CST, Danvers, USA |
| IRF3 | Proteintech, Rosemont, USA |
| GPX6 | Cloud-Clone, Houston, USA |
| CAT | Proteintech, Rosemont, USA |
| HO-1 | Proteintech, Rosemont, USA |
| SOD1 | Proteintech, Rosemont, USA |
| NRF1 | Proteintech, Rosemont, USA |
| NRF2 | Proteintech, Rosemont, USA |
| p-mTOR | Proteintech, Rosemont, USA |
| mTOR | Proteintech, Rosemont, USA |
| IFNβ | Santa cruz, Texas, USA |
| GAPDH | ZSGB-BIO, Beijing, China |
| β-actin | Proteintech, Rosemont, USA |
| Anti-rabbit | CST, Danvers, USA |
| Anti-mouse | Proteintech, Rosemont, USA |
| Anti-rat | Beyotime, Shanghai, China |

Table S2. Sequences of genes detected in real-time PCR.

| **Genes** | **Sequences** |
| --- | --- |
| *Pgam5*  (mouse) | F- GAACTACATCCACCGAGCTGA  R- GGGAAACTGCAACGCTCTAC |
| *β-actin*  (mouse) | F- ACGGCCAGGTCATCACTATTG  R- CAAGAAGGAAGGCTGGAAAAGA |
| *Col1a1*  (mouse) | F- CACCTGGTCCACAAGGTTTC  R- CCCATCATCTCCATTCTTGC |
| *Col3a1*  (mouse) | F- TGCTGGAAAGGATGGAGAGT  R- TGGGCCTTTGATACCTGGAG |
| *Acta2*  (mouse) | F- AGGCACCACTGAACCCTAAG  R- GACAGCACAGCCTGAATAGC |
| *Timp1*  (mouse) | F- CATGGAAAGCCTCTGTGGAT  R- CTCAGAGTACGCCAGGGAAC |
| *Mmp9*  (mouse) | F- AAGGCAGCGTTAGCCAGAAG  R- GCGGTACAAGTATGCCTCTGC |
| *Mmp12*  (mouse) | F- TGTGACTGTACCAAGCCATCA  R- TGTGCTGGCTAGAGAATGCTT |
| *Mmp13*  (mouse) | F- GGAGCCCTGATGTTTCCCAT  R- GTCTTCATCGCCTGGACCATA |
| *Tgfb*  (mouse) | F- CGCAACAACGCCATCTATGA  R- ACTGCTTCCCGAATGTCTGA |
| *Tnfa*  (mouse) | F- GGCCTCCCTCTCATCAGTTC  R- CACTTGGTGGTTTGCTACGA |
| *Il1*  (mouse) | F- TGTGAAATGCCACCTTTTGA  R- GGTCAAAGGTTTGGAAGCAG |
| *Il6*  (mouse) | F- CAAAGCCAGAGTCCTTCAGAG  R- GAGCATTGGAAATTGGGGTA |
| *Il10*  (mouse) | F- CAGAGCCACATGCTCCTAGA  R- GCTTGGCAACCCAAGTAACC |
| *Arg1*  (mouse) | F- CAAGACAGGGCTCCTTTCAG  R- TGAGTTCCGAAGCAAGCCAA |
| *Nos2*  (iNOS, mouse) | F- AATCTTGGAGCGAGTTGTGG  R- CAGGAAGTAGGTGAGGGCTTG |
| *F4/80*  (mouse) | F- TGACTCACCTTGTGGTCCTAA  R- CTTCCCAGAATCCAGTCTTTCC |
| *Cd68*  (mouse) | F- TAGGACCGCTTATAGCCCAAG  R- GTGGTGGCAGGGTTATGAGT |
| *Ly6c*  (mouse) | F- GCAGTGCTACGAGTGCTATGG  R- ACTGACGGGTCTTTAGTTTCCTT |
| *Mpo*  (mouse) | F- AGTTGTGCTGAGCTGTATGGA  R- CGGCTGCTTGAAGTAAAACAGG |
| *Gpx6*  (mouse) | F- GTCACGGTTTTGGGCTTTCC  R- GTCACGGTTTTGGGCTTTCC |
| *Beta-actin*  (human) | F-CATGTACGTTGCTATCCAGGC  R-CTCCTTAATGTCACGCACGAT |
| *Cat*  (human) | F-CTCCGGAACAACAGCCTTCT  R-ATAGAATGCCCGCACCTGAG |
| *Ho-1*  (human) | F- CTGCTGACCCATGACACCAA  R- TCCACGGGGGCAGAATCTTG |
| *Nrf2*  (human) | F- AGGTTGCCCACATTCCCAAA  R- ACGTAGCCGAAGAAACCTCA |
| *Sod1*  (human) | F- AGGCATGTTGGAGACTTGGG  R- TGCTTTTTCATGGACCACCAG |
| *Mmp12*  (human) | F- TTTTTGCCCGTGGAGCTCAT  R- TCGAAATGTGCATCCCCTCC |
| *Timp*  (human) | F- CATCCGGTTCGTCTACACCC  R- TCCTGCAGTTTTCCAGCAATG |
| *Mmp13*  (human) | F- CAGTTTGCAGAGCGCTACCT  R- TTCTCGGAGCCTCTCAGTCA |
| *Pgam5*  (human) | F- GCTACATCGTGTGCAGAGCA  R- TCTTGTCGGGAGGCATGAAC |

Table S3. Sources of ELISA kit used in cytokines assay.

| **ELISA kit** | **Manufacturer** |
| --- | --- |
| human IL-1β | Dakewe, Guangdong, China, Cat#: 1110122 |
| human IL-6 | Dakewe, Guangdong, China, Cat#: 1110602 |
| human IL-10 | Dakewe, Guangdong, China, Cat#: 1111002 |
| human TNF-a | Dakewe, Guangdong, China, Cat#: 1117202 |

**Additional Figures**


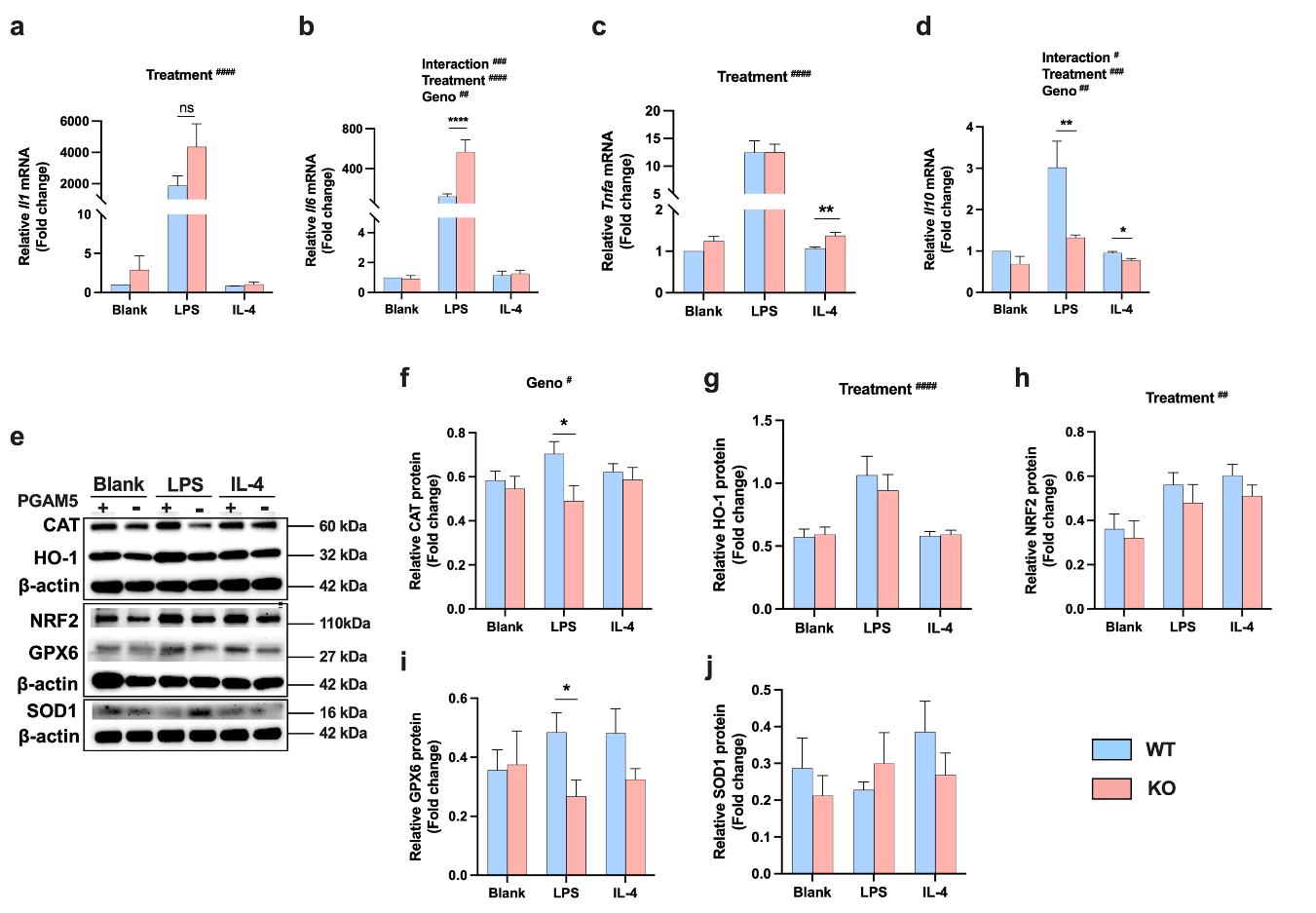


Figure S1: Knockout of PGAM5 increased mRNA expressions of pro-inflammatory genes and decreased protein levels of antioxidative genes in BMDMs.

a-d: Relative mRNA expressions of pro-inflammatory cytokines including IL-1 (a) , IL-6 (b), TNF-α (c) and IL-10 (d) measured by real-time PCR in BMDMs. e-j: Relative protein levels of CAT (f), HO-1 (g), NRF2 (h), GPX6 (i) and SOD1 (j) measured by Western blot. ^*^p<0.05, ^**^p<0.01, ^****^p<0.0001, statistical differences between WT and KO group. ^#^p<0.05, ^##^p<0.01, ^###^p<0.001, ^####^p<0.0001, statistical differences between treatments, genotypes or their interactions. ns, no significance; LPS, lipopolysaccharide; CAT, catalase; HO-1, heme oxygenase-1; NRF2, nuclear factor erythroid 2- related factors; GPX6, glutathione peroxidase 6; SOD1, superoxide dismutase 1; WT, wild type; KO, knockout.


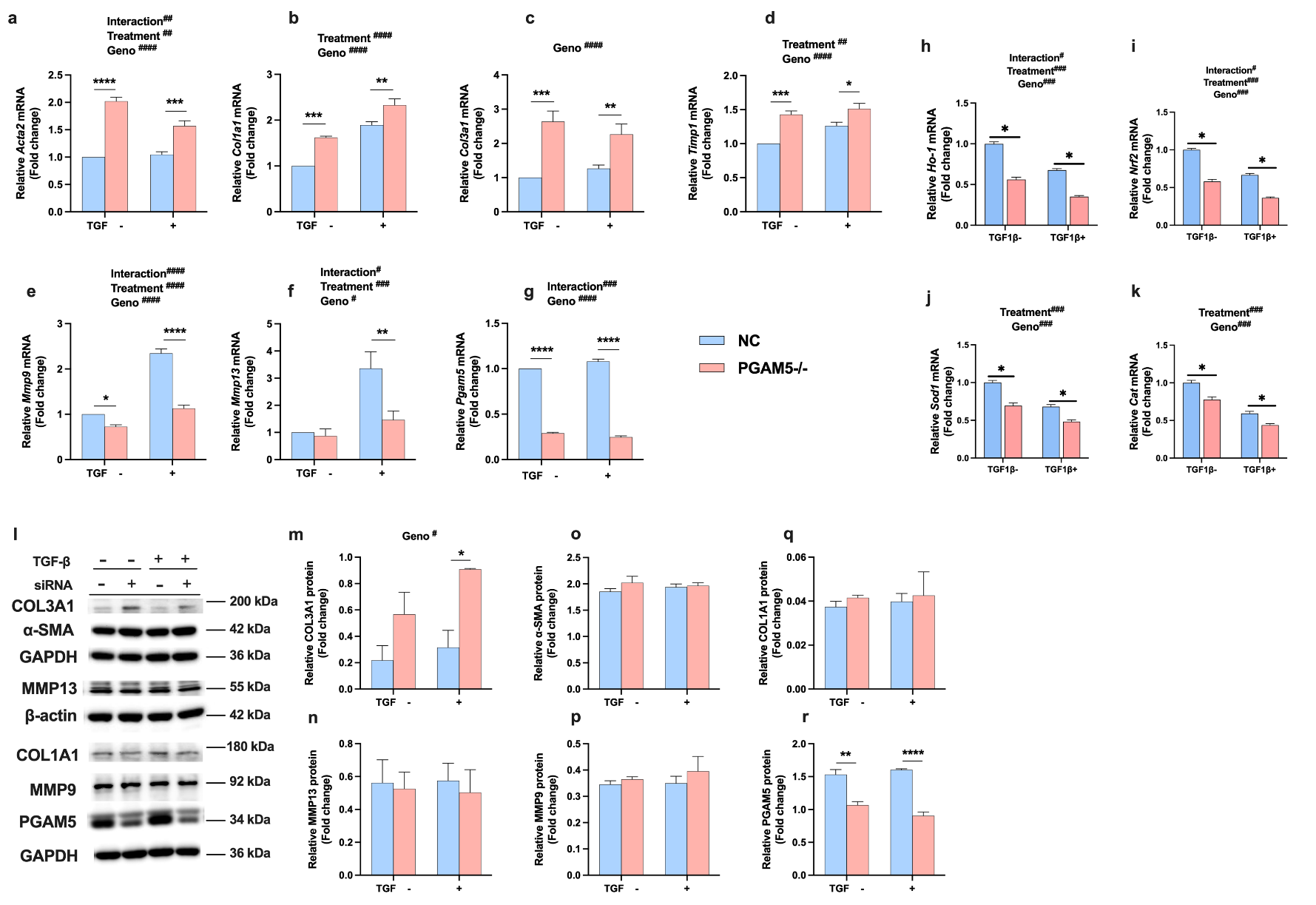


Figure S2: Knockdown of PGAM5 increased expressions of pro-fibrotic and decreased mRNA expressions of antioxidative genes in LX2 cells.

a-g: Relative mRNA expressions of pro-fibrotic genes including Acta2 (a) , Col1a1 (b), Col3a1 (c), Timp1 (d) Mmp9 (e) and Mmp13 (f), and mRNA expressions of PGAM5 (g) were shown. h-k: Relative mRNA expressions of antioxidative genes including Ho-1 (h) , Nrf2 (i), Sod1 (j) and Cat (k) were shown. l-r: Relative protein levels of COL3A1 (m), MMP13 (n), α-SMA (o), MMP9 (p), COL1A1 (q) and PGAM5 (r) were detected in LX2 cells. ^*^p<0.05, ^**^p<0.01, ^***^p<0.001, ^****^p<0.0001, statistical differences between NC and PGAM5^-/-^ group. ^#^p<0.05, ^##^p<0.01, ^###^p<0.001, ^####^p<0.0001, statistical differences between treatments, genotypes or their interactions. COL, collagen; Mmp, matrix metalloproteases; Timp1, tissue inhibitors of Mmps; Ho-1, heme oxygenase-1; Nrf2, nuclear factor erythroid 2- related factors; Sod1, superoxide dismutase 1; Cat, catalase; NC, negative control; PGAM5^-/-^, PGAM5-knockdown.


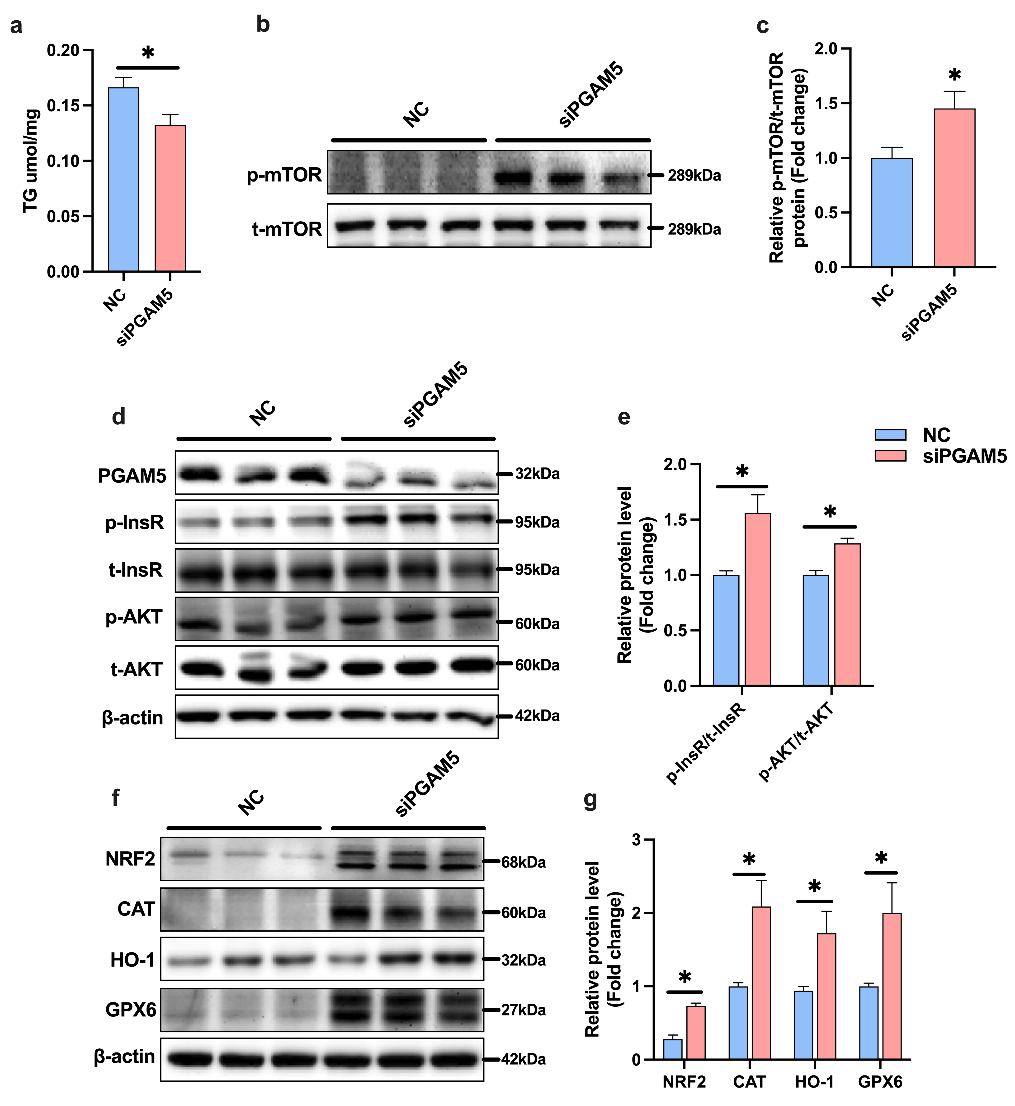


Figure S3: Knockdown of PGAM5 reduced TG accumulation and up-regulated antioxidative genes in 3T3L1 cells.

a: TG levels measured in adipocytes. b-g: Relative protein levels of p-mTOR/t-mTOR (b-c), proteins of insulin transduction pathway including p-InsR/t-InsR and p-AKT/t-AKT (d-e) and antioxidative proteins including NRF2, CAT, HO-1 and GPX6 (f-g) were measured in 3T3L1 cells.

^*^p<0.05, statistical differences between NC and siPGAM5 group. mTOR, mammalian target of rapamycin; InsR, insulin receptor β; AKT, protein kinase B; NRF2, nuclear factor erythroid 2- related factors; CAT, catalase; HO-1, heme oxygenase-1; GPX6, glutathione peroxidase 6; NC, negative control; siPGAM5, PGAM5 knockdown.


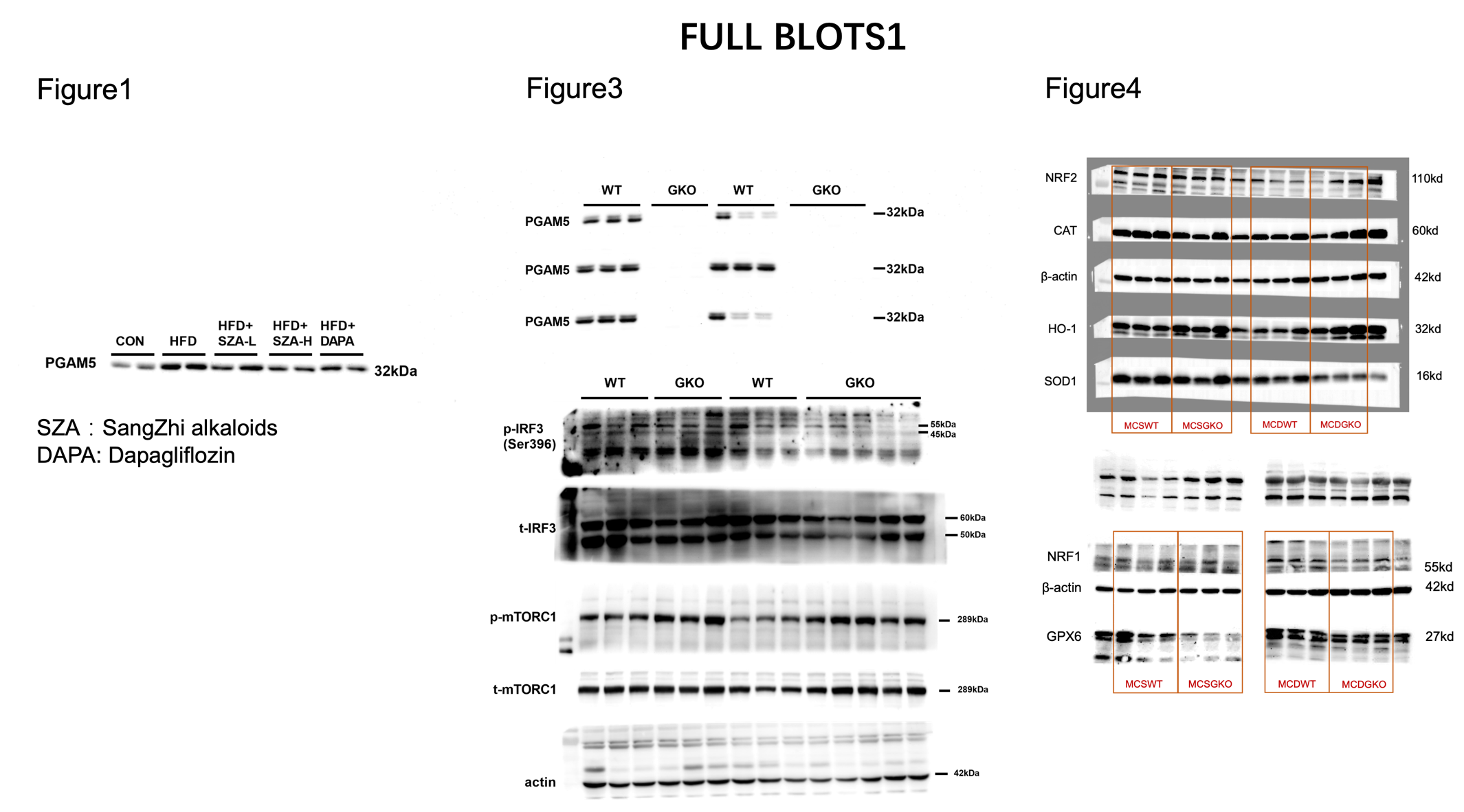


Figure S4: Original blots for Figs. 1-4.


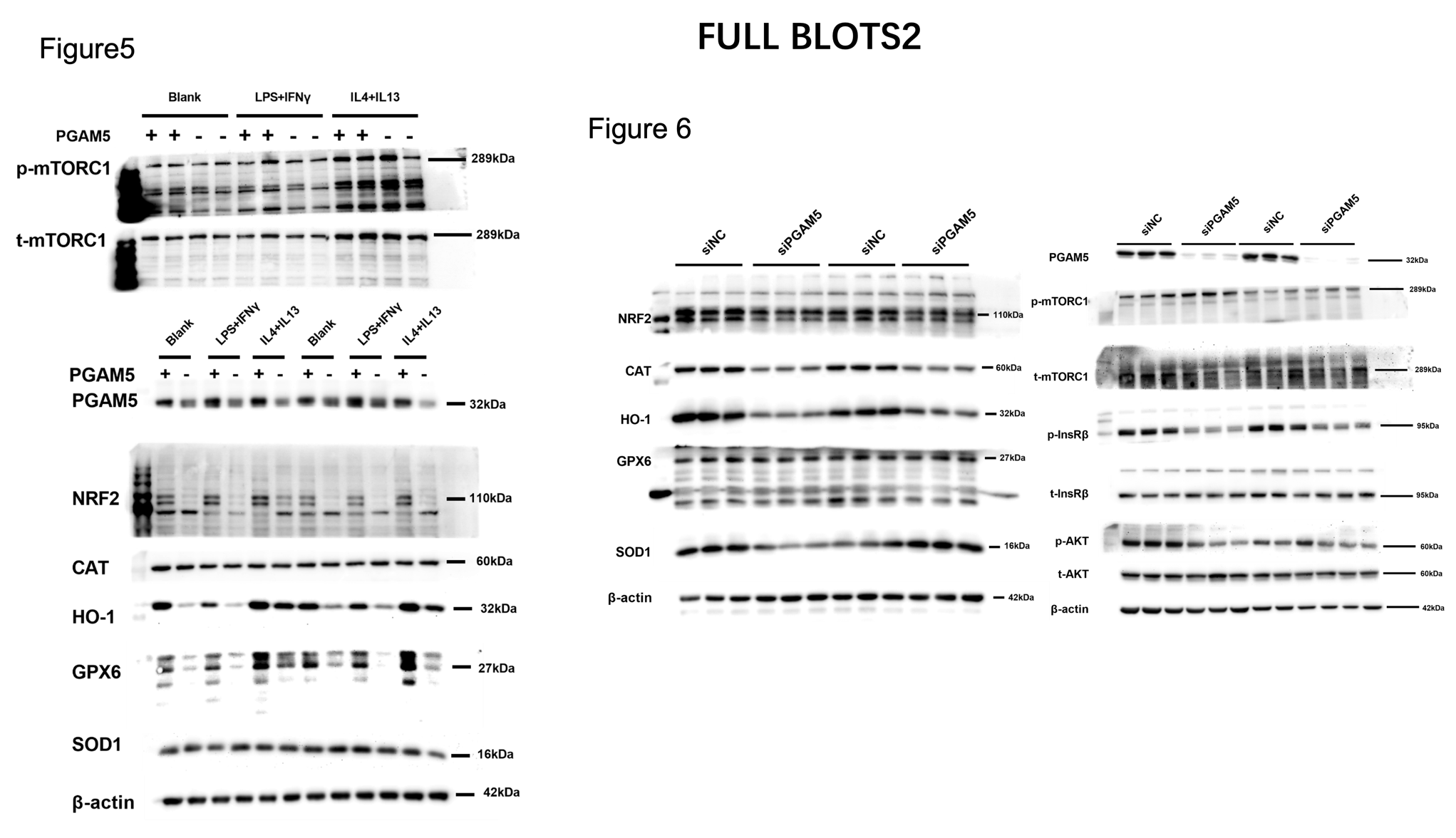
Figure S5: Original blots for Figs. 5, 6.
